# Reducing inequalities through greater diversity in clinical trials – As important for medical devices as for drugs and therapeutics

**DOI:** 10.1016/j.conctc.2025.101467

**Published:** 2025-03-05

**Authors:** Laurence S.J. Roope, Jessica Walsh, Maddie Welland, Gabrielle Samuel, Heidi Johansen-Berg, Anna C. Nobre, Stuart Clare, Helen Higham, Jon Campbell, Tim Denison, Karla L. Miller, Seena Fazel, Matthew L. Costa, Andrew Farmer, Marian Knight, Rachel Taylor, Lorna R. Henderson, Angeli Vaid, John Geddes, Vasiliki Kiparoglou, Helen McShane, Philip M. Clarke

**Affiliations:** a Health Economics Research Centre, Nuffield Department of Population Health, University of Oxford, Oxford, United Kingdom; b National Institute for Health Research Oxford Biomedical Research Centre, John Radcliffe Hospital, Oxford, United Kingdom; c National Institute for Health and Care Research Oxford Health Biomedical Research Centre, Oxford, United Kingdom; d Wellcome Centre for Integrative Neuroimaging, FMRIB, Nuffield Department of Clinical Neurosciences, University of Oxford, Oxford, United Kingdom; e Wellcome Centre for Human Genetics, Nuffield Department of Medicine, University of Oxford, Oxford, United Kingdom; f Department of Experimental Psychology, University of Oxford, Oxford, United Kingdom; g Nuffield Department of Clinical Neurosciences, University of Oxford, Oxford, United Kingdom; h Department of Psychiatry, University of Oxford, Oxford, United Kingdom; i Oxford Trauma and Emergency Care, Kadoorie Centre, Nuffield Department of Orthopaedics, Rheumatology and Musculoskeletal Sciences, University of Oxford, Oxford, United Kingdom; j Nuffield Department of Primary Health Care Sciences, University of Oxford, Oxford, United Kingdom; k National Perinatal Epidemiology Unit, Nuffield Department of Population Health, University of Oxford, Oxford, United Kingdom; l Radcliffe Department of Medicine, University of Oxford, Oxford, United Kingdom; m The Griffin Institute, Northwick Park & St Mark's Hospital, Y Block, Watford Road Harrow, HA1 3UJ, United Kingdom; n Nuffield Department of Medicine, University of Oxford, Oxford, United Kingdom; o Centre for Health Policy, Melbourne School of Population and Global Health, University of Melbourne, Melbourne, VIC, Australia

**Keywords:** Diversity, Efficient trial design, Inequality, Levelling up, Trial recruitment, Underserved

## Abstract

In medicine and public health, the randomised controlled trial (RCT) is generally considered the key generator of ‘gold standard’ evidence. However, basic and clinical research and trials are often unrepresentative of real-world populations. Recruiting insufficiently diverse cohorts of participants in trials (e.g. in terms of socioeconomic status, racial and ethnic background, or sex and gender) may not only overstate the general effectiveness of a technology; it may also actively increase health inequalities. We highlight some general issues in this domain, before discussing several specific illustrative examples in the context of medical devices. High quality evidence on factors that would improve trial recruitment is extremely limited. There is a clear need for research on candidate strategies for improving recruitment of under-represented groups in RCTs. These could include, for example, offering various forms of financial incentives; non-monetary incentives, such as preferential access to the technologies that are being tested if they are found to be effective; and various types of informational messages and nudges; as well as involvement of community partners and champions in the recruitment process. Ideally, recruitment practices should ultimately be based on evidence generated from RCTs. Studies Within a Trial (SWAT), where randomised experiments are built into the actual recruitment processes in RCTs, are an ideal way to gain this evidence. SWAT studies are seeing an increase in traction, as indicated by funding streams in bodies such as the UK-based NIHR. Making greater funding available for studies of this kind is needed to improve the evidence base on how best to improve diversity in trial recruitment.

## 1 Introduction

In medicine and public health, the randomised controlled trial (RCT) is generally considered the key generator of ‘gold standard’ evidence. In this perspective, we argue that trial recruitment is an issue that warrants greater consideration in clinical and public health RCTs, because of the potentially serious consequences from not having sufficiently diverse and representative study samples. Diversity in RCTs made some brief headlines during the COVID-19 pandemic. As people around the world waited eagerly for news in the race for effective vaccines, US trials of a promising candidate vaccine developed by Moderna had to be slowed down due to the under-representation of Black, Latino and Native American participants [1]. Lack of diversity was also documented in other COVID-19 vaccine trials [2]. Yet insufficient diversity in RCT participation, and indeed health and care research generally [3,4], is a much broader issue. As we discuss below, it is a problem that not only threatens the generalisability of many RCTs, but potentially also exacerbates health inequalities in society, leaving population subgroups both ‘unrepresented’ and ‘underserved’ (the latter term reflecting the sense that the research community should provide them a better service) [3,4].

This issue is now highlighted in guidance from the World Health Organisation on conduct of clinical trials [5], and the National Institute for Health and Care Research (NIHR) in the United Kingdom. Thus, it is increasingly recognised, but the extent of impact is not widely appreciated and the ways it can be addressed not well articulated. We begin by highlighting some general issues in this domain, before discussing several specific illustrative examples in the context of medical devices; the latter draw on our recent submission to a UK-government-commissioned independent review on equity in medical devices [6].

## 2 General issues regarding inclusive recruitment into randomised controlled trials

Greater focus is needed in clinical and public health RCTs on the extent to which they are generalizable, beyond the specific context in which the evidence is generated (e.g. Deaton and Cartright [7]). It is well known that basic and clinical research and trials are often unrepresentative of real-world populations [8]. Important subgroups that are often under-represented include, among others: people of low socioeconomic status (SES), ethnic and racial minorities, women, pregnant women, people with cognitive impairment, non-cisgender people and sexual minorities [9]. There is also an issue of global imbalance, in that participants from low-income countries are generally under-represented, and often not represented [10]. Yet it is important to recognise that whether a group is under-served can also be context-specific, depending on the population that research aims to serve. For example, while women are under-represented in RCTs across a wide range of diseases [11], men have been found to be under-represented in, for example, weight loss trials [12].

In many clinical and public health interventions, heterogeneous treatment effects, correlated with such socio-demographic factors, are plausible [13]. For example, there is evidence that some psychiatric treatments can be less effective for people with lower SES [14]. In terms of real world effectiveness, if issues such as adherence and prescribed use of a particular health technology play a critical role, possible heterogeneous treatment effects by SES should also be considered in data analyses [15].

Specific health technologies have been shown to be less effective, ineffective, or even unsafe among a subgroup of the population that they aim to serve, as in the case of some pulse oximeters. If this subgroup is under-represented in trials of such technologies [16], there are several major implications. First, the expected value of the average treatment effect (ATE) estimate from the trial sample will be higher than the true population level ATE. Thus, on average, over repeated runs of such a trial, the treatment will appear more effective on average than will actually be the case when scaled up to population level. Second, if the technologies are found to be effective in the trial sample, but the trial is not powered to assess effectiveness in under-represented subgroups, they will likely be deemed a broad success and used widely in the real world, including by subgroups for whom they are not effective and potentially even unsafe or damaging. To make matters worse, when supposedly ‘effective’ treatments are found, there may be less incentive for future research, and future chances of finding treatments that are effective for under-represented groups may actually decrease. In this way, recruiting insufficiently diverse cohorts of participants in trials (e.g. in terms of SES, racial and ethnic background, or sex and gender) may not only overstate the general effectiveness of a technology; it may also actively increase health inequalities.

A number of general recommendations have been made in the academic literature on ways to reduce barriers to participation of underserved groups in trials [3,5,6,17,18]. These include, among others, approaches to overcoming financial, language and educational/health literacy barriers; inflexible working arrangements [18,19]; and issues of trust [20,21]. Each of these barriers may need to be tackled in multiple ways. For instance, while financial barriers might be reduced through higher participant compensation and prompt and comprehensive payment of expenses, it may be equally important to design studies with minimal in-person visits and to ensure that, whenever visits are essential, medical notes are provided in good time to employers. Language can be a major barrier to recruiting ethnic minorities, immigrants, and people with limited education and/or health-literacy. As well as translating study materials, such as consent forms and trial brochures, into patients’ first languages, it may also be valuable to employ interpreters. More generally, all such study materials, should be written in a highly accessible way, with inclusion in mind [17], aiming for a recommended reading age of no more than 11–12 [22], a target that appears to be widely unmet [23]. Similar principles apply to advertising materials designed to promote recruitment. Beyond the language used in advertising trial opportunities, it is important that the advertising is targeted widely so that it reaches broad populations. In many cases, it would also be valuable to engage directly with specific cultures and local communities, such as certain religious groups, to discuss a given research project. Taking the time needed to build and maintain effective community partnerships may be needed to overcome issues relating to trust [20]. There is also evidence to suggest that it would be beneficial to involve under-represented groups at an early stage, while trials are being designed [24,25]. Among other things, considering inclusion at an early stage can help to ensure that eligibility criteria and recruitment pathways do not unintentionally limit participation [17].

Drawing on our recent submission to the UK-government-commissioned independent review on equity in medical devices [6], in what follows, we outline a number of specific examples where lack of diversity in RCTs has sometimes been problematic, and where research efforts are underway to address this. In supplementary materials, we provide some broader examples from our submission, on other aspects of the research and development process, aside from RCTs, where greater diversity in participation would be valuable.

## 3 Improving equity in medical devices through better diversity in participation in RCTs: some real-life examples

### 3.1 Pulse oximeters

Pulse oximeters are perhaps the most well-known illustration of the potential dangers of involving an insufficiently diverse patient pool in medical device development. A number of studies have shown that for patients with darker skin, pulse oximeters may over-estimate their true oxygen level. In turn, this may lead to an under-estimation of the severity of patient illness, inadequate treatment and unnecessary harm. There is currently a study underway [26], embedded within a broader RCT, that aims to determine whether skin tone affects the accuracy of a variety of different pulse oximeters and confirm whether they over-estimate the level of oxygen in people with darker skin. To do this, the study is comparing the values from pulse oximeters to precise oxygen levels measured in blood samples, in patients with a range of different skin tones. The results will provide information that will allow manufacturers to adapt how pulse oximeters work in the future.

### 3.2 Orthopaedic implants

People with cognitive impairment are often excluded from hip fracture trials. This is a concerning omission as approximately 40 % of all patients with hip fracture have some degree of cognitive impairment on or during their admission to hospital [27]. More can be done to include cognitively impaired patients in trials. For example, a number of RCTs (including Fernandez et al., 2022 [27]) have been embedded within the “WHiTE” cohort, which facilitates the inclusion of patients with cognitive impairment. After extensive consultation with patients and their carers, a process was developed that allows patients to participate with the support of someone engaged in caring for them or with an interest in their welfare or, in the rare circumstance where no carer is available, with another nominated consultee, such as an independent healthcare professional [28].

### 3.3 Electroencephalogram (EEG) recording of brain activity

There is evidence from a number of studies that the design of EEG electrodes is not compatible with thick, curly hair common to many people of African ethnicity [[29], [30], [31]]. EEG electrodes require secure, direct and long-lasting electrode-to-scalp contact, which means that specific hair characteristics, such as texture and density, affect electrode placement and decrease the signal to noise ratio. This has led to a significant exclusion of Black participants from clinical research and is a barrier to accessing EEG treatment clinically [29].

Research initiatives are underway to deploy EEG systems in multiple low- and middle-income countries, where these hair characteristics are particularly prevalent. EEG systems are relatively low-cost, but hairstyle has been a major impediment to deployment, and solutions are currently being investigated by the Oxford Martin Programme on Global Epilepsy [32].

Emerging technologies that are designed to leverage coarse and curly hair and hairstyles, such as the Sevo clip, are being developed and trialled [30]. Guidance should be developed that can be used to inform patients or research participants on compatible hairstyles and preparation requirements for a successful scan [33,34]. This may include the development of a styling guide, in collaboration with Afro-hairstylists, that can be taken to a regular hair stylist. It should be noted that this does lead to additional financial and time burdens for which an allowance should be considered to remove/reduce these barriers.

### 3.4 Medical devices used in children

Medical devices used in children are often repurposed from adult devices, which are more economically viable due to the larger market. When repurposed for children, there are implications that need to be considered, other than simply size. These can include growing with the device, changes in heart rate, respiratory rate and blood pressure as children grow, maximal infection deterrence and acceptability to children and their families. Thorough testing should therefore be done in the paediatric population that the device is expected to work in. See, for example, Piper et al., 2022 [35] in the context of neurostimulation devices.

Developers should ensure that a broad range of implications of repurposing a device from adults to children are considered, and that devices are specifically tested in children's populations, as is being carrying out in the CADET trial [36]. Alternatively, developers of devices should consider specifically developing devices for children.

Devices for children should also include specific guidance for how they should be used in children, with reference to specific child characteristics (such as growth, longevity), so that it is clear that these have been thought about and appropriately tested.

## 4 Improving the evidence base on promoting trial participation in under-represented groups

A Cochrane review on strategies to improve overall trial recruitment generally – not only recruitment of minorities – found that ‘high-certainty evidence’ on factors that promote recruitment is extremely limited [37]. We argue that there is a clear need for research on candidate strategies for improving recruitment of under-represented groups in RCTs. These could include, for example, offering various forms of financial incentives (on which there is currently ‘moderate-certainty evidence [37]); non-monetary incentives, such as preferential access to the technologies that are being tested if they are found to be effective; and various types of informational messages and nudges; as well as involvement of community partners and champions in the recruitment process [38]. Importantly, it is to be anticipated that optimal recruitment strategies may differ substantially by context. For example, within a given population subgroup, what motivates a healthy person to be vaccinated against a disease that is unlikely to affect them is likely to differ markedly from what motivates someone with cancer to participate in a trial that might alter the disease's course.

While a variety of qualitative and quantitative evidence can inform recruitment strategies, ideally practices should ultimately be based on evidence generated from RCTs. Studies Within a Trial (SWAT), where randomised experiments are built into the actual recruitment processes in RCTs, are an ideal way to gain such evidence [[39], [40], [41]]. To aid comparability across such studies, and potentially facilitate subsequent meta-analyses, it may be valuable to include some form of standardised reporting for socio-demographic characteristics in these studies, including ethnicity and indicators of SES. Indeed, it is worth considering whether standardised reporting of socio-demographic characteristics, encompassing more information than the usual sex and age reporting, should be mandatory in trials of health technologies. In so doing, care should be taken to avoid lumping distinct meaningful groups, which may have their own particular needs and health profiles, into an ‘other’ category – as is a widespread issue in the context of sex, gender and sexuality [9]. As well as facilitating analysis of possible heterogeneous treatment effects, and illuminating health inequalities, this would complement and add value to the growing trend of documenting and analysing reasons for trial participation refusal [42]. SWAT studies are seeing an increase in traction, as indicated by the increasing numbers of SWATs detailed on the Online Resource for Research in Clinical triAls (ORRCA) database [43,44], which organises and maps the current literature on recruitment and retention research. It is also indicated by the emergence of funding streams in bodies such as the UK-based NIHR [45]. Ultimately, making greater funding available for studies of this kind is needed to improve the evidence base on how best to improve diversity in trial recruitment.

## 5 Conclusion

In this perspective, we have argued that insufficient diversity in recruitment not only threatens the generalisability of many RCTs, but potentially also exacerbates health inequalities in society. It is a problem that has had much less attention from clinical and population health researchers than it deserves. While far from exhaustive, the examples we have outlined illustrate that lack of diversity in research and development has an adverse impact across a wide range of different types of health technologies. However, there is much that can be done to address these issues, with roles for all those involved in the production of research, including regulators and funders.

## CRediT authorship contribution statement

**Laurence S.J. Roope:** Writing – review & editing, Writing – original draft, Project administration, Conceptualization. **Jessica Walsh:** Writing – review & editing. **Maddie Welland:** Writing – review & editing. **Gabrielle Samuel:** Writing – review & editing. **Heidi Johansen-Berg:** Writing – review & editing. **Anna C. Nobre:** Writing – review & editing. **Stuart Clare:** Writing – review & editing. **Helen Higham:** Writing – review & editing. **Jon Campbell:** Writing – review & editing. **Tim Denison:** Writing – review & editing. **Karla L. Miller:** Writing – review & editing. **Seena Fazel:** Writing – review & editing. **Matthew L. Costa:** Writing – review & editing. **Andrew Farmer:** Writing – review & editing. **Marian Knight:** Writing – review & editing. **Rachel Taylor:** Writing – review & editing. **Lorna R. Henderson:** Writing – review & editing. **Angeli Vaid:** Writing – review & editing. **John Geddes:** Writing – review & editing. **Vasiliki Kiparoglou:** Writing – review & editing. **Helen McShane:** Writing – review & editing, Funding acquisition. **Philip M. Clarke:** Writing – review & editing, Supervision.

## Ethics approval and consent to participate

Not applicable.

## Consent for publication

Not applicable.

## Availability of data and materials

Not applicable.

## Funding

This article was funded by the National Institute for Health Research (NIHR) Oxford Biomedical Research Centre (BRC). LSJR, VK, AF, HMcS and PMC are supported by the NIHR Oxford Biomedical Research Centre, Oxford. LSJR and PMC are supported by the NIHR Oxford Health Biomedical Research Centre, Oxford. KLM is supported by the Wellcome Trust (203139/Z/16/Z, 224573/Z/21/Z, 215573/Z/19/Z). The Wellcome Centre for Integrative Neuroimaging is supported by core funding from the Wellcome Trust (203139/Z/16/Z and 203139/A/16/Z). MLC, AF, MK, JG and HMcS are NIHR Senior Investigators. The views expressed are those of the authors and not necessarily those of the NHS, the NIHR or the Department of Health and Social Care. The study funders had no role in the design of the study, in the writing of the paper or in the decision to submit for publication.

## Declaration of competing interest

The authors declare that they have no known competing financial interests or personal relationships that could have appeared to influence the work reported in this paper.

## Data Availability

No data was used for the research described in the article.

## Abbreviations

ATE: Average Treatment EffectCOVID-19: Coronavirus disease 2019
EEG: Electroencephalogram
RCT: Randomised Controlled Trial
SES: Socio-economic Status
