# Supplementary material for: Reducing inequalities through greater diversity in clinical trials – As important for medical devices as for drugs and therapeutics
Source: Contemp Clin Trials Commun. 2025 Mar 5;45:101467. doi: 10.1016/j.conctc.2025.101467 (PMC11999327; doi:10.1016/j.conctc.2025.101467)
Supplement: Multimedia component 1 [file mmc1.docx]

**Supplementary Materials**

**Examples from other areas of research and development, beyond RCTs, where greater diversity in participation would be valuable.**

*Magnetic resonance imaging (MRI)*

Some of the safety settings for MRI scanners are based on entering sex, yet it is not clearly defined as to what the appropriate sex is to put for someone who is transgender or intersex. This should be considered in the development of MRI scanners.

Currently, ‘sex assigned at birth’ is typically used as the sex entered on MRI scanners for transgender or intersex people. However, it is generally unknown whether this compromises patient safety or scanner performance, as there are no recommendations from manufacturers and no guidance available.

MRI scanners, and other devices that are reliant on sex, should ensure that the design and development accounts for transgender and intersex individuals. The UKCA mark legislation should ensure that any device that relies on a sex/gender input has been appropriately tested in those who do not necessarily clearly identify with one sex. This emphasises the importance of ensuring that research participation data on sex and gender are captured using sufficiently nuanced categories [9].

MRI scanner manufacturers, and other devices that are reliant on sex, should ensure they provide guidance for device operators on how to safely use the device on someone who is transgender or intersex, ensuring that safety is not compromised.

*Medical Device Interfaces*

Medical and monitoring device interfaces are poorly standardised and unregulated in their presentation of data across all healthcare settings. In a pending publication [46], a pre-study survey determined that there is no consistency in equipment provision, which is likely to represent a risk in most large organisations. Staff that move frequently between sites and organisations are constantly having to adapt to new equipment.

A significant proportion of current NHS England staff are non-native English speakers (EU nationals 5.4%, non-EU nationals 9.2% as of March 2021). Device development and testing does not explicitly consider ease of use for staff without English as a first language or with any visual impairment (e.g. colour-blindness, which reduces ability to discern differences between certain colours on screen). Testing phases for device interfaces should involve a wide variety of users to account for any barriers to accessibility.

Adequate monitoring is an essential standard of care in anaesthesia and critical care. During emergencies a great deal of attention is focused towards monitoring devices, and these are situations in which task-demands, time pressure, and mental workload are highest. It is essential that clinicians can acquire information accurately and rapidly.

Poor standardisation can lead to false reassurance when encountering a new device. If it shares generic features with equipment that staff have used previously, they may feel they are fully competent in using it, without recognising important differences.

A high proportion of errors in critical care occur due to failings around monitoring. This is directly correlated to the poor standardisation, inaccessibility and poor design of medical and monitoring device interfaces.

The pending data referred to above [46], and the wider psychology literature, provide some recommendations in equipment provision. Specific visual cueing (i.e. searching something that has been seen before) allows best performance, so parameter position and colour schemes should be standardised across clinical areas.

Ideally, displays should be limited only to information of immediate relevance, although the increasing availability of touch-screen devices may afford opportunities to place secondary information on tabs that remain hidden until required. New automatic devices or smart-monitoring systems might offer further opportunities to display information dynamically.

Positioning of items on the screen affects their likelihood of being noticed. Parameters should be organised linearly to facilitate left-to-right scanning wherever possible; critical values should always be displayed topmost. If confirmatory information derived from other sources must be displayed on the same screen, this secondary information should be placed below the primary source.

*Clinical risk tools/algorithms*

It is very important to include a comprehensive sample in the development of clinical tools/algorithms. Problems with these types of devices are rare in datasets that involve national populations. When they are used in other countries, local validations can be done to test their accuracy in this new population. When trained on databases that under-represent certain groups, algorithms develop biased predictions that are not applicable for those populations that they were not trained on (i.e., those groups for which there is under representation). For example, there was a machine learning dermatology app that used data from the International Skin Imaging Collaboration (ISIC) - an open-source repository of skin images from predominantly fair-skinned populations around the world. As a result of under-representation, the app was not trained to diagnose skin conditions as effectively on darker skin [47]). Machine learning algorithms trained on Eurocentric genomic datasets will be vulnerable to similar biases. In the field of genomics, studies have demonstrated the difficulties of classifying variants in people from populations that are less represented in datasets globally and the increased likelihood that they would either be misclassified [48] or classified as variants of unknown significance (VUS) [49,50].

If high quality methods are used in the development of clinical risk tools and algorithms, any problems can be substantially mitigated. These methods include large comprehensive datasets, such as population-based ones, and also careful and adequately powered validations. It is important that any validations present a full range of performance measures that include how models discriminate (separate out risk groups) and are calibrated (how predicted risks compare with observed ones). Too often, they present only one or two of such measures, such as an overall measure of how well a model separates out high from low risk, but fail to include whether the predictions are accurate (e.g. what high risk actually means for an individual outcome over a specified time period) beyond such broad risk groups. Following reporting guidelines in prognostic medicine, such as TRIPOD guidelines, are key (https://www.equator-network.org/reporting-guidelines/tripod-statement/).

Good quality methods are also key. The OxMIS risk calculator was developed for assessment of suicide risk in people with severe mental illness [51]. It was developed using a national population dataset, reported a full range of performance statistics, published the algorithm in full, and followed recommended guidelines for best methods in the fields of prognostic medicine.

Use of ethnicity, as a variable, can be justified if it leads to a clear improvement in the performance of any clinical algorithm and hence potentially reduce adverse outcomes in the ethnic group in question. One example is that the QRisk algorithm uses ethnicity to predict risk of cardiovascular events, which is defensible as there is high quality research showing that certain ethnic groups are at higher risk.

*Health and safety standard markings*

In many countries, standard markings are used to signify that products, including some health technologies, have been assessed to meet some level of health, safety and/or environmental standards – for example CE marking in the European Economic Area and Turkey, and the UKCA in the UK. In the context of some types of health technologies, including pulse oximeters and many other medical devices, such marks could be used to ensure that manufacturers have adequately considered the diverse patient populations that will be using the technology, and have ensured sufficient representation in trials. Information about safety testing that has been conducted, and any implications for health technology use in any subgroups of the population, should also be made clear in the instructions/guidance that comes with such technologies.

**Supplementary References**

46. Grieg, Higham and Nobre, 2022. Manuscript in preparation.

47. Lashbrook A. AI-driven dermatology could leave dark-skinned patients behind. The Atlantic. 2018 Aug;16.

48. Manrai AK, Funke BH, Rehm HL, Olesen MS, Maron BA, Szolovits P, Margulies DM, Loscalzo J, Kohane IS. Genetic misdiagnoses and the potential for health disparities. New England Journal of Medicine. 2016 Aug 18;375(7):655-65.

49. Kurian AW, Ward KC, Hamilton AS, Deapen DM, Abrahamse P, Bondarenko I, Li Y, Hawley ST, Morrow M, Jagsi R, Katz SJ. Uptake, results, and outcomes of germline multiple-gene sequencing after diagnosis of breast cancer. JAMA oncology. 2018 Aug 1;4(8):1066-72.

50. Caswell-Jin JL, Gupta T, Hall E, Petrovchich IM, Mills MA, Kingham KE, Koff R, Chun NM, Levonian P, Lebensohn AP, Ford JM. Racial/ethnic differences in multiple-gene sequencing results for hereditary cancer risk. Genetics in Medicine. 2018 Feb;20(2):234-9.

51. Fazel S, Wolf A, Larsson H, Mallett S, Fanshawe TR. The prediction of suicide in severe mental illness: development and validation of a clinical prediction rule (OxMIS). Translational psychiatry. 2019; 9(1):1-10.
